# Predictors of Major Adverse Cardiovascular Events in Stable Patients After ST Elevation Myocardial Infarction

**DOI:** 10.3390/clinpract15060106

**Published:** 2025-05-30

**Authors:** Lidija Savic, Damjan Simic, Ratko Lasica, Gordana Krljanac, Sanja Stankovic, Igor Mrdovic, Milika Asanin

**Affiliations:** 1 Faculty of Medicine, University of Belgrade, 11000 Belgrade, Serbiagkrljanac@gmail.com (G.K.); masanin2013@gmail.com (M.A.); 2 Cardiology Intensive Care Unit & Cardiology Clinic, Emergency Hospital, University Clinical Center of Serbia, 11000 Belgrade, Serbiaigormrd@gmail.com (I.M.); 3 Center for Medical Biochemistry, Emergency Hospital, University Clinical Center of Serbia, 11000 Belgrade, Serbia

**Keywords:** stable post myocardial infarction, major adverse cardiovascular events, prognosis

## Abstract

Background/aim: The aim of this study was to determine predictors of major adverse cardiovascular events, including MACE (mortality, non-fatal recurrent infarction, non-fatal stroke, and target vessel revascularization-TVR) in stable post-STEMI patients. Method: We analyzed STEMI patients without cardiogenic shock at admission included in our STEMI Register. The patients were treated with primary PCI. The follow-up period was eight years. Results: From 1 December 2006 to 31 December 2016, a total of 3079 patients were included in the Register. In the first year, MACE was registered in 348 (11.3%) patients. The remaining patients were considered stable. They were included in further analysis. At eight years, the rates were as follows: MACE 3.9%, non-fatal recurrent infarction 2.1%, TVR 1.8%, non-fatal stroke 0.5%, and mortality 2.1%. Predictors for 8-year MACE were age >60 years (60–69 vs. <60 years HR 1.65; 70–79 vs. <60 years HR 1.82; ≥80 vs. <60 years HR 3.16), EF < 50% (EF 40–49% HR 2.38; EF < 40% HR 2.32), diabetes mellitus (HR 1.49), and 3-vessel coronary artery disease (HR 1.44). Conclusions: Four predictors identified stable post-STEMI patients who remained at a higher risk for the occurrence of MACE. Stable post-STEMI patients with one or more of these risk factors may require more aggressive secondary prevention measures or a personalized approach to improve their prognosis.

## 1. Introduction

Primary percutaneous coronary intervention (pPCI), dual antiplatelet therapy, increasing expertise, and improvements in secondary prevention have significantly reduced mortality and the occurrence of adverse events in patients with ST-elevation myocardial infarction (STEMI), as compared to the fibrinolytic era [1,2,3,4,5,6,7,8,9,10,11,12,13]. Improved in-hospital survival has led to a growing population of post-STEMI patients [2,4,14]. The highest rate of mortality and non-fatal adverse events after hospital discharge in STEMI patients treated with pPCI is in the first months of the first year after the index event. After this period, the incidence of mortality and other adverse events declines. It has been shown in the literature that the overall long-term mortality in patients who had experienced STEMI and were treated with pPCI is similar to the mortality in the general population (adjusted for age and sex) [1]. At the same time, other studies have shown that the recovery of the same life expectancy, as that in the general population, was registered only in STEMI patients after they had survived the first 30 days [5,14].

In the literature, patients with myocardial infarction (MI), for whom not a single adverse event was registered during the first year following the index event, are called stable post-MI patients and are generally considered low-risk patients [4,7,14]. On the other hand, there are data on some stable post-MI patients with certain risk factors, i.e., in whom the risk for adverse events remains high in the long-term follow-up [6,7,10,14,15].

Most studies analyzing prognosis after MI published so far included patients with STEMI and non-STEMI (NSTEMI). In some of these studies, myocardial revascularization was not performed in all patients, and the long-term prognosis was analyzed in the entire follow-up after the index event (including the first months, i.e., the first year after the index event), while other articles analyzed data from randomized studies [1,2,4,5,16,17,18].

One year upon STEMI, the majority of patients remain only on single antiplatelet therapy; partial attenuation of ischemic risk provided by pharmacological therapy may be lost, and follow-up examinations are often thinned out (which especially applies to patients who did not have a single adverse ischemic event during the first year). Therefore, it could be important to analyze the incidence of adverse events and define predictors for their occurrence in stable post-STEMI patients, which may help in better risk stratification and a personalized approach in terms of secondary prevention and/or further follow-up. We hypothesize that baseline characteristics and characteristics during hospitalization for index events may predict the occurrence of adverse events in stable post-STEMI patients.

This study aims to analyze the incidence and determine predictors of major adverse cardiovascular events (MACE) across eight-year follow-ups in stable patients treated with primary PCI after STEMI.

## 2. Materials and Methods

### 2.1. Study Population, Inclusion and Exclusion Criteria, Data Collection, and Definitions

In the present study, we analyzed STEMI patients, hospitalized between 1 December 2006, and 31 December 2016, who were included in the prospective University Clinical Center of Serbia STEMI Register. The purpose of the prospective University Clinical Center of Serbia STEMI Register has already been published elsewhere [19]. The objective of the Register is to gather data on the management and short- and long-term outcomes of patients with STEMI treated with pPCI. All consecutive STEMI patients, aged 18 years or older, within 12 h after the onset of symptoms and who were admitted to the Coronary Care Unit after being treated with pPCI in the Catheterization Lab of the Center, were included in the Register. All involved patients received written information about their participation in the Register and the long-term follow-up, and their verbal and written consent was obtained [19]. Only patients with cardiogenic shock at admission were excluded from this Register (hypotension with systolic blood pressure ≤90 mmHg with signs of hypoperfusion and need for iv inotropes. Patients presenting with heart failure Killip class II and III and patients who developed cardiogenic shock later during hospitalization were included in the Register. For the purpose of this study, patients who died or had other adverse events in the first 12 months were excluded. Patients without adverse events in the first 12 months were considered stable and were included for further analysis.

**Figure 1:**
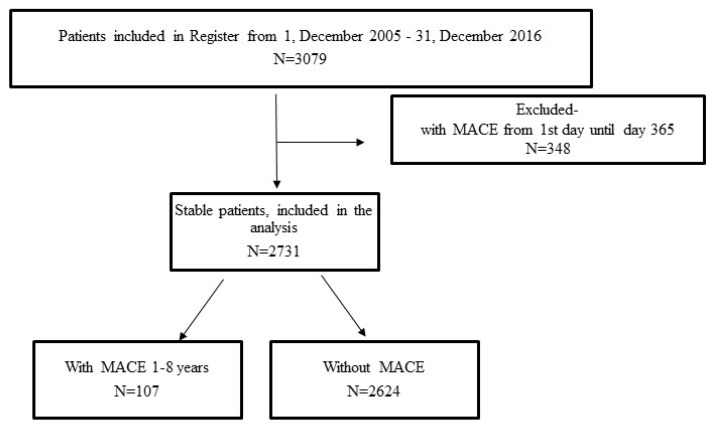
The flowchart of patient selection. MACE = major adverse cardiovascular events.

The study enrolment criteria (patient selection) are presented in Figure 1.

Coronary angiography, primary PCI, and stenting of the infarct-related artery (IRA) were performed using the standard technique. The femoral approach was the preferred vascular access in patients hospitalized between 2006 and 2012, while in patients hospitalized between 2013 and 2016, the radial approach was the preferred vascular access. Loading doses of aspirin (300 mg), clopidogrel (600 mg), or ticagrelor (180 mg) were administered to all patients before pPCI, depending on current guideline recommendations and current practice at the time of the index procedure. Selected patients were also given the GP IIb/IIIa receptor inhibitor during the procedure. After pPCI, patients were treated according to the current guidelines.

Demographic, baseline clinical, laboratory, angiographic, and procedural data were collected and analyzed. In the first three days after intervention (pPCI), an echocardiographic examination was performed on patients. The left ventricular EF was assessed using the biplane method. The value of EF > 50% was considered preserved EF; the value of EF 40–49% was considered moderately reduced EF; and the value of EF < 40% was considered severely reduced EF [20]. Baseline kidney function (blood for creatinine analysis was drawn at hospital admission before pPCI and iodine contrast administration) was assessed using the Modification of Diet in Renal Disease (MDRD) equation, and the value of the estimated glomerular filtration rate (eGFR) below 60 mL/min/m^2^ was considered to represent chronic kidney disease (CKD). Anemia at admission was defined as the baseline hemoglobin level of <120 g/L in men and <110 g/L in women.

Patients were followed up at eight years after enrolment into the Register (from the date of pPCI). Follow-up data were obtained through telephone interviews and outpatient visits. We analyzed the occurrence of MACE, which included overall mortality, nonfatal infarction, nonfatal stroke, and target vessel revascularization (TVR). The information about death was obtained from death certificates or discharge forms (if the patient had previously been hospitalized).

Non-fatal recurrent myocardial infarction was defined according to the Fourth Universal Definition of Myocardial Infarction [21]. TVR was defined as ischemia-driven percutaneous revascularization of the target vessel performed due to restenosis or other complications. Stroke was defined as a new onset of focal or global neurological deficit lasting more than 24 h. Computed tomography (CT) was used to diagnose (ischemic) stroke. The Emergency Hospital neurologist was responsible for diagnosing and treating stroke [19].

### 2.2. Ethics

The study protocol was approved by the Ethics Committee of the University of Belgrade, Faculty of Medicine (approval No. 470/II-4, 21 February 2008). The study was conducted in keeping with the principles outlined in the Declaration of Helsinki. Written informed consent was obtained from all patients for their participation in the Register.

### 2.3. Statistical Analysis

Categorical variables were expressed as frequency and percentage, and continuous variables were expressed as the median (med), with 25th and 75th quartiles (IQR). Analysis for normality of data was performed using the Kolmogorov–Smirnov test. Baseline differences between groups were analyzed using the Man–Whitney test for continuous variables and the Pearson χ^2^ test for categorical variables. Multiple missing data imputation was performed for (maximal) troponin level (1.9% missing), glycemia at admission, creatinine at admission leukocytes and hemoglobin at admission (0.5–1% missing). The Kaplan–Meier method was used for constructing the probability curves for eight-year cumulative adverse cardiovascular events. The Cox proportional hazard model (backward method, with *p* < 0.10 for entrance into the model) was used to identify univariable and multivariable predictors for the occurrence of composite adverse events. All variables that differed in preliminary analysis between stable post-STEMI patients with and without MACE were included in the Cox regression model. Receiver operating characteristics (ROC) curves were used for determining the cut-off value for the age that was the most powerful in predicting the occurrence of MACE and determining the model’s predictive accuracy (area under the curve-AUC) over time. Bootstrapping with 1000 resamples was applied for internal validation of predictors. Two-tailed *p*-values of <0.05 were considered statistically significant. We used the SPSS statistical software, Version 19, for statistical analysis (SPSS Inc., Chicago, IL, USA).

## 3. Results

### 3.1. MACE Incidence in the First 12 Months

Between 1 December 2006, and 31 December 2012, a total of 3079 patients with STEMI undergoing pPCI without cardiogenic shock at admission were included in Register. In the first 365 days after the index event (in-hospital and after hospital discharge), MACE was registered in 348 (11.3%) patients; 201 (6.53%) patients died (among whom 132 (4.8%) died in hospital); 43 (1.4%) patients had a non-fatal recurrent MI; 11 (0.4%) patients had a non-fatal stroke; and TVR was performed in 100 (3.2%) patients (Figure 1).

### 3.2. Patient Characteristics and MACE Incidence in Stable Post-STEMI Patients

The 2731 patients without MACE in the first 12 months after the index event were defined as stable post-STEMI population.

Baseline demographic, clinical, laboratory, echocardiographic, angiographic characteristics, and ongoing therapy in all stable STEMI patients and stable patients with and without MACE are shown in Table 1.

The mean age of the entire cohort was 58 (51, 67) years. In total, 728 (26.7%) patients were female. As compared with patients without MACE, patients with MACE were older; they were more likely to have previously suffered a stroke, to have diabetes mellitus, multivessel coronary artery disease on the initial angiogram, higher glycemia at admission, a higher troponin level, and lower EF. There was no significant difference regarding ongoing medication (at day 366) among patients with and without MACE.

**Figure 2:**
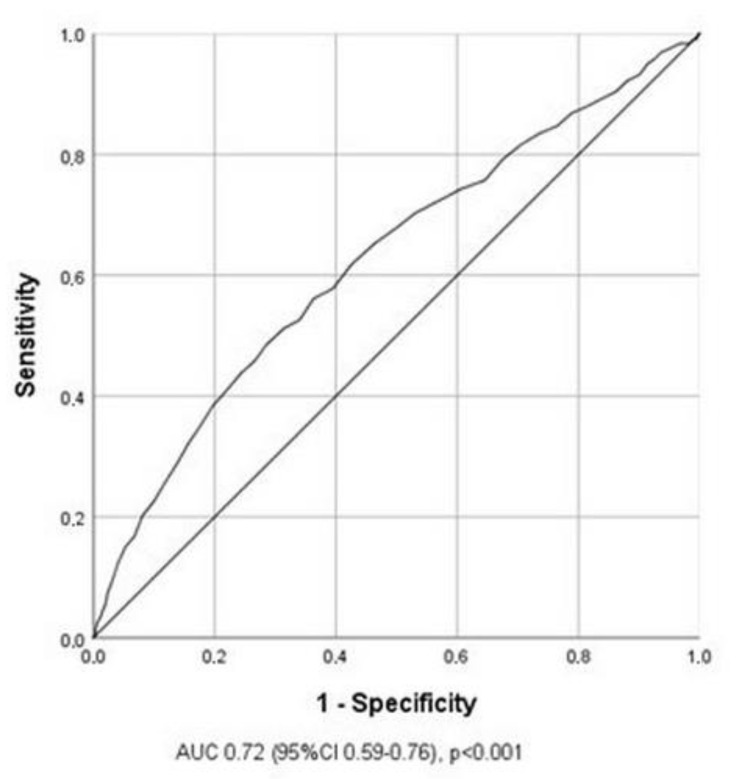
Receiver operating curve (ROC) analysis for defining the cut-off value for age.

ROC curve analysis revealed that 60 years was the most powerful predictive age for the occurrence of the analyzed composite CV events, as shown in Figure 2.

The incidence of MACE and adverse events included in composite MACE are shown in Table 2.

**Figure 3:**
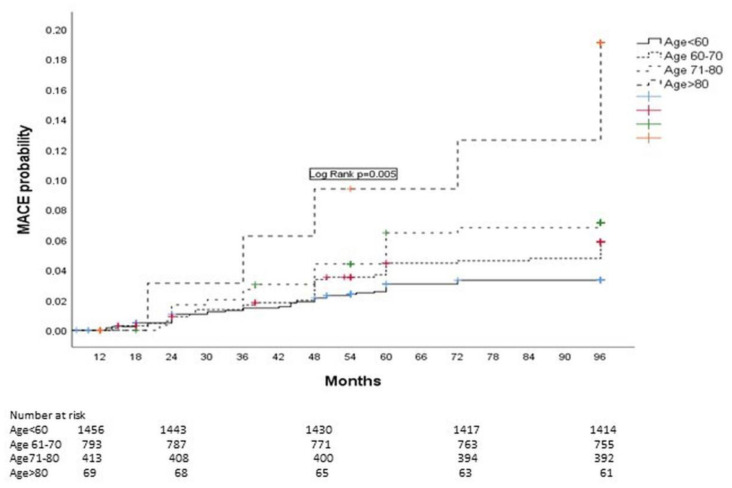
Kaplan–Meier curve showing MACE probability during an 8-year follow-up stratified by age.

Kaplan–Meier curves showing MACE probability in the whole cohort, stratified by age, are shown in Figure 3

Cardiovascular causes of death (including fatal recurrent infarction, fatal stroke, sudden death, and progression of heart failure) were registered in 38 patients (66.6% of all deaths). The remaining 19 patients (18.9% of all deaths) died from non-cardiovascular causes (such as cancer, pneumonia, and dementia), *p* < 0.001. In-stent restenosis or very late stent thrombosis were the causes of non-fatal reinfarction in 19 (42.2%) patients, while new coronary artery stenosis/occlusion was the cause of non-fatal reinfarction in 26 (58.8%) patients.

### 3.3. Predictors for the Occurrence of MACE in Stable Post-STEMI Patients

Multivariable analysis identified four independent predictors of MACE during 8-year follow-ups: age >60 years (and the risk increased with every 10-year increase in age), reduced EF < 50% (the risk increased with declining EF), the presence of 3-vessel disease, and diabetes mellitus.

Univariate and multivariate Cox regression analyses showing predictors for MACE during eight-year follow-ups are shown in Table 3.

Sensitivity, specificity, positive predictive value (PPV), and negative predictive value for each predictor are shown in Table 4.

**Figure 4:**
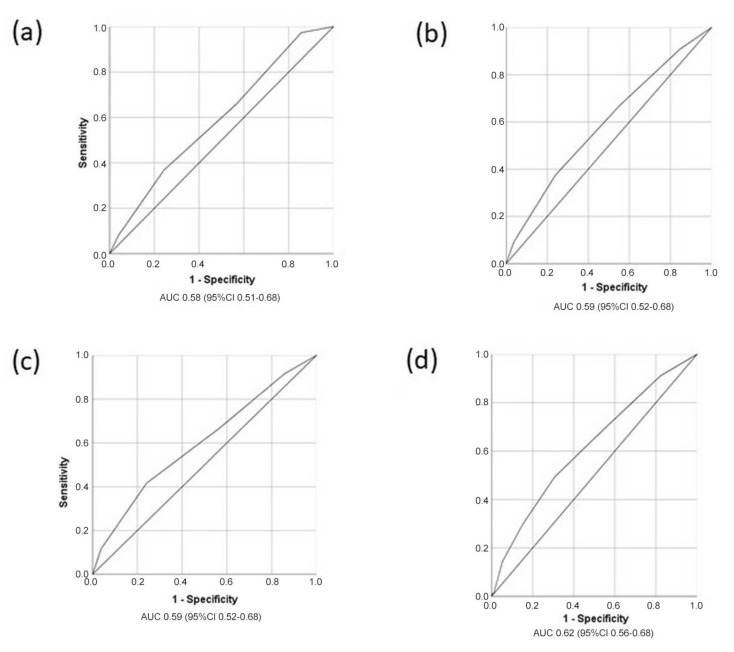
Receiver operating characteristics (ROC) curves showing the model’s discriminatory characteristics at 2 years (curve (**a**)), 4 years (curve (**b**)), 6 years (curve (**c**)), and 8 years (curve (**d**)).

The model’s discriminative performance over time (at 2, 4, 6, and 8 years) is shown in Figure 4.

**Figure 5:**
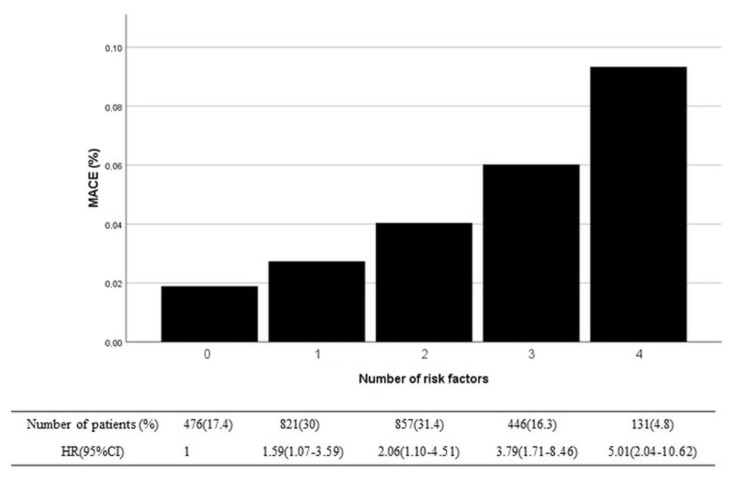
Observed MACE rates and the risk for MACE associated with the presence of risk factors. MACE = major adverse cardiovascular events.

Predicted MACE probability, the event rate, and HR for the occurrence of MACE in relation to the presence of none to four risk factors in stable post-STEMI patients are shown in Figure 5.

## 4. Discussion

Our study showed the highest incidence of MACE in STEMI patients treated with pPCI in the first year after the index event. During further follow-up of up to eight years, the incidence of MACE in stable patients was lower than in the first year and gradually increased, without excess. Cardiovascular causes of mortality were significantly higher than non-cardiovascular causes of mortality. Also, the appearance of new lesions on the coronary arteries was a more frequent cause of non-fatal recurrent infarction than restenosis or very late stent thrombosis. Independent predictors for the occurrence of MACE in stable post-STEMI patients during eight-year follow-ups were well-known predictors for the occurrence of MACE in the entire STEMI population, i.e., (older) age, reduced EF, diabetes mellitus, and 3-vessel coronary artery disease.

### 4.1. Patient Characteristics, the Incidence of MACE, and MACE Predictors in Stable Post-STEMI Patients

To date, there have been a number of trials reporting long-term mortality after pPCI, but there have not been many studies analyzing long-term prognosis in stable post-STEMI patients treated with pPCI against which we could directly compare our results. Our findings are in keeping with previous reports that have found generally favorable long-term prognosis in STEMI patients treated with pPCI [1,5,14]. However, in the stable patients in our study, the incidence of MACE was still lower than in the aforementioned studies. There could be several explanations for this finding. One could be that, compared to other studies, the basal and clinical characteristics of our post-STEMI patients were more favorable—our patients were generally younger than the patients analyzed in other studies and had fewer comorbidities and a lower prevalence of multivessel coronary disease. Also, in these studies, cardiogenic shock at admission was not mentioned as the exclusion criteria. Since older patients and patients with some well-known high-risk features for adverse events (such as older age, comorbidities, multivessel disease, heart failure, previous cardiovascular disease, etc.) usually experience these adverse events during the first years after MI, it was not surprising that our stable post-STEMI patients had better baseline characteristics as compared with patients from previous studies wherein the occurrence of adverse events after the day of the infarction was predominantly analyzed [1,4,5,8,14,21,22,23,24,25]. Another explanation could be related to the fact that our stable patients were found to be more likely to receive guidelines-based therapy than reported in many previous studies [4,5,10,14,22]. Also, a lower incidence of MACE might be expected in reports from single-center studies with a higher rate of revascularization in MI patients and with a closer clinical follow-up program [7,25] than in the reports that included data from multiple centers or national databases. It is well known that residual risk of mortality and MACE remains higher in patients who were not treated invasively [2,7,26].

In a study by Pascual et al., it was shown that the survival rate observed during a four-year follow-up in STEMI patients surviving the first 30 days after the index event was similar to our findings. Furthermore, patients surviving the first year had a life expectancy similar to that of the general population of the same age, sex, and geographical region [5,27]. In a study by Brogan et al., it was found that the 5-year prognosis in patients with STEMI treated with pPCI was excellent, i.e., that the overall 5-year survival was similar to that of the general population. In this study, overall 5-year mortality was examined and stable patients were not separately analyzed [1]. In a study by Chen et al., the incidence of composite cardiovascular (CV) outcomes (recurrent MI, stroke, and death) during a three-year follow-up in stable patients with STEMI and NSTEMI was analyzed. The incidence of composite CV events in the first year was similar to our findings; however, the incidence of composite CV events as well as the incidence of the individual adverse events after the first 12 months analyzed in this study were higher than in our patients. As compared to our study, in the cited study, the patients were older, the prevalence of comorbidities was higher, and myocardial revascularization during the index event was performed only in about 65% of stable patients and in about 56% of the patients with adverse events. Also, a smaller percentage of patients received guidelines-based medication during follow-up, as compared to our patients. Nevertheless, several predictors of analyzed composite CV events were consistent with the predictors from our study [4]. In a study by Jernber et al., it was found that the risk for the occurrence of subsequent cardiovascular events in stable patients after MI remained high, with one in five patients experiencing an event during subsequent years [22]. In this study, all patients with MI were included; they were, on average, older than our patients, and revascularization during the index event was performed in about 50% of the cases. Patients who did not undergo revascularization at the index event had an elevated risk of future CV events, as compared with revascularized patients; i.e., in this study, no revascularization was an independent predictor of combined endpoint within the first year or after a year of follow-up [22]. In a study by Özcan et al., the long-term risk in stable post-MI patients was analyzed. The main finding was that the risk for recurrent cardiovascular events after 365 days remained equally as high as in the first 365 days. Coronary artery disease severity remained the most critical factor for recurrent cardiovascular events both before and after the first 365 days, while one of the independent predictors was the absence of revascularization at the index event, which was performed in around 65% of the cases in the subgroup of stable post-STEMI patients [7]. In a study by Mureddu et al., the 5-year prognosis was analyzed in patients with MI who had no adverse events in the first 30 days after the index event. It was found that the incidence of major adverse cardiovascular events during the 5-year follow-up was 24% in patients with uncomplicated MI and up to 75.5% in patients who had heart failure and characteristics for high thrombotic risk. However, in this study, in addition to patients with MI type 1, patients with a secondary diagnosis of MI defined as any concomitant MI complicated within primary diagnosis were also included, which reduced survival and increased the risk of complications [24]. Finally, a systematic review by Johansson highlights large information gaps occurring one year or more after the index MI. MI (STEMI and NSTEMI) survivors who remained at high risk after the first year were older individuals and those with hypertension or diabetes [6], which is also in keeping with our findings.

### 4.2. Clinical and Future Implications

Our findings may add to the existing knowledge about long-term prognosis in STEMI patients treated with contemporary primary PCI. Although stable post-STEMI patients treated with pPCI have a generally favorable prognosis, we should not forget that some of them remain at higher risk of adverse events in the long-term follow-up. The risk of future ischemic events and death is compounded by the patient’s baseline characteristics, treatment at the index event, and the management practice both at discharge and subsequently [2]. As expected, mortality increased with age. Age is a risk factor for atherosclerosis development and prognosis. Older patients usually have more comorbidities as compared with younger patients, and this can negatively influence their prognosis following MI [6]. Reduced EF is a marker of extensive myocardial necrosis and worse left ventricular systolic function [28]. Multivessel disease is an independent predictor of adverse left ventricular remodeling and impaired EF in STEMI patients [29]. Diabetes is not only a risk factor for the development but also for the progression of coronary artery disease, as well as a marker of worse prognosis after STEMI. Patients with DM have complex metabolic and neurohumoral disturbances, including dyslipidemia with the presence of small, dense LDL particles, which are extremely atherogenic end products with high levels of glycosylation that can cause a proinflammatory state and the progression of atherosclerosis [30,31]. The findings that our patients died predominately from cardiovascular causes and that the appearance of new lesions on the coronary arteries was a more frequent cause of non-fatal recurrent infarction than restenosis or very late stent thrombosis are in concordance with defined independent predictors for MACE. Clinicians should recognize the presence of these high-risk characteristics because patients with one or more high-risk features may benefit from more aggressive secondary prevention measures and/or more frequent follow-ups even after a year has elapsed after the infarction [6,10,16,25]. Some of more aggressive secondary prevention measures might include better glycemic control and introducing SGLT2 inhibitors, better blood pressure control, and aggressive treatment of dyslipidemia with higher doses of statin and/or a combination of statin and ezetimibe. Inclusion in cardiac rehabilitation programs should also be considered. Also, extending the duration of dual antithrombotic therapy beyond the first year may be considered according to the presence of some high-risk characteristics for the recurrent ischemic events, keeping in mind current guidelines recommendation [6,32].

### 4.3. Study Limitations

This study should be viewed in the context of its limitations. This is a single-center study. It is observational, but it is controlled, prospective, and has included all consecutive STEMI patients, limiting possible selection bias. Patients with cardiogenic shock at admission were excluded from our Register. There are no data on follow-up echocardiographic examinations to show whether there has been a certain degree of recovery or deterioration in the left ventricular systolic function. We did not use other measures for determining systolic function, such as myocardial deformation imaging. However, many cornerstone clinical trials to date have used EF to stratify patients [31,33]. The study was not designed to evaluate whether changing pharmacological treatment during follow-up would have an impact on the long-term outcome in the analyzed patients.

## 5. Conclusions

In the analyzed stable post-STEMI patients treated with pPCI, the incidence of subsequent major adverse cardiovascular events in long-term follow-up of up to eight years was low. An increased risk of the occurrence of major adverse cardiovascular events was found in patients older than 60 years, patients with EF < 50%, patients with three vessel coronary disease, and diabetic patients. Stable post-STEMI patients with one or more of these risk factors may require more aggressive secondary prevention measures or a personalized treatment approach to improve their prognosis.

## Figures and Tables

**Table 1:** Baseline clinical, laboratory, angiographic, procedural characteristics, and concomitant therapy in stable patients after STEMI, with or without MACE in the 8-year follow-up.

Characteristics	All Stable Patients *N* = 2731	With MACE *N* = 107	Without MACE *N* = 2624	*p* Value
Age, years med (IQR)	60 (51, 67)	62 (53, 71)	59.5 (50, 67)	<0.001
Age < 60 years, *n* (%)	1456 (53.3)	40 (38)	1416 (53.9)	0.005
Age 61–70 years, *n* (%)	793 (20.9)	38 (36.2)	755 (28.7)	0.011
Age 71–80 years, *n* (%)	413 (15.1)	21 (20)	392 (14.9)	0.010
Age >80 years, *n* (%)	69 (2.5)	8 (7.5)	61 (2.3)	0.001
Female, *n* (%)	728 (26.7)	24 (22.8)	704 (26.8)	0.369
BMI, med (IQR)	26.5 (24.5, 29.6)	26.8 (24.9, 28.7)	26.4 (24.8, 29.8)	0.638
Previous MI, *n* (%)	252 (9.2)	14 (13.3)	238 (9.1)	0.124
Previous angina, *n* (%)	185 (6.8)	8 (7.6)	177 (6.7)	0.726
Previous stroke, *n* (%)	94 (3.4)	9 (8.6)	85 (3.3)	0.003
DM, *n* (%)	499 (18.3)	30 (28.6)	469 (17.8)	0.005
Hypertension, *n* (%)	1789 (65.5)	77 (73.3)	1712 (65.2)	0.225
HLP, *n* (%)	1665 (60.1)	69 (65.7)	1596 (60.8)	0.309
Smoking, *n* (%)	1530 (56)	54 (51.4)	1476 (56.2)	0.606
Family hystory, *n* (%)	1939 (70.1)	29 (27.6)	910 (34.6)	0.137
Pain duration, hours med (IQR)	2.5 (1.5, 4)	3 (1.5, 4)	2.5 (1.5, 4)	0.807
New or presumably new onset atrial fibrillation, *n* (%) *	149 (5.5)	11 (10.5)	138 (5.2)	0.021
Complete AV block at admission, *n* (%)	101 (3.4)	6 (5.7)	95 (3.6)	0.526
BBB at admisson ECG, *n* (%) **	128 (4.7)	7 (6.1)	121 (4.5)	0.861
Killip class >1, at admission, (%)	260 (9.5)	11 (10.5)	249 (9.5)	0.977
Systolic BP at admission, med (IQR)	140 (120, 150)	140 (110, 155)	135 (120, 150)	0.610
Heart rate at admission med (IQR)	77 (66, 88)	72 (69, 83)	80 (70, 90)	0.415
Multivessel disease, *n* (%)	1486 (54.4)	67 (63.8)	1419 (54)	0.041
3-vessel disease, *n* (%)	674 (24.7)	36 (34.3)	638 (24.9)	0.020
LM stenosis, *n* (%)	158 (4.1)	6 (5.9)	152 (5.7)	0.973
Postprocedural flow TIMI <3, *n* (%)	83 (3)	2 (1.9)	81 (3.1)	0.498
Stent implanted, *n* (%)	2707 (99.1)	102 (97.2)	2605 (99.2)	0.987
Subacute stent thrombosis, *n* (%)	12 (0.04)	1 (0.9)	11 (0.4)	0.422
Glicoprotein IIb/IIIa inhibitor, *n* (%)	967 (35.4)	30 (28.5)	937 (35.7)	0.151
CK MB max, med (IQR)	1826 (980, 3308)	2276 (1113, 3549)	1816 (906, 3315)	0.067
eGFR, med (IQR)				
Troponin max, med (IQR)	34 (10, 97.1)	48 (16, 150)	31 (10, 93)	0.009
Anemia at admission, *n* (%)	198 (7.2)	12 (11.4)	186 (7.1)	0.082
WBC count at admission, med (IQR)	11.3 (9.2, 13.6)	10.5 (8.8, 13.5)	11.1 (9.2, 13.6)	0.950
Glycemia at admission, mmol/L med (IQR)	7.2 (5.9, 9)	7.6 (6.1, 11)	7.1 (5.9, 8.9)	0.010
CKD, *n* (%)	1831 (67)	74 (70.6)	1801 (68.6)	0.672
EF, med (IQR)	50 (40, 55)	45 (40, 55)	50 (45, 55)	<0.001
EF < 40%, *n* (%)	278 (10.3)	15 (14.3)	263 (10)	<0.001
EF 40–49%, *n* (%)	977 (35.7)	59 (56.2)	918 (34.9)	<0.001
EF ≥ 50%, *n* (%)	1476 (54)	31 (29.5)	1445 (55)	<0.001
Ongoing medication at day 366 after index event ***				
Aspirin, *n* (%)	2697 (98.7)	99 (94.2)	2598 (98.9)	0.567
Beta blockers, *n* (%)	2587 (94.7)	93 (88.5)	2494 (94.7)	0.268
ACE inhibitors/ARB, *n* (%)	2413 (88.3)	88 (83.8)	2325 (88.5)	0.813
Statin, *n* (%)	2577 (94.4)	95 (90.4)	2482 (94.5)	0.990
Diuretic, *n* (%)	499 (18.3)	72 (68.6)	427 (16.2)	0.002
Calcium antagonist, *n* (%)	103 (3.8)	4 (3.8)	99 (3.8)	0.909
OAC, *n* (%)	30 (1.1)	3 (2.8)	27 (1.1)	0.622
Amiodarone, *n* (%)	81 (2.9)	8 (7.6)	73 (2.8)	0.003

MACE = major adverse cardiovascular event; med = median; IQR= interquartile range; BMI = body mass index; MI = myocardial infarction; DM = diabetes mellitus; EF = left ventricular ejection fraction; HLP = hyperlipidemia; AV = atrioventricular; BBB = bundle branch block on ECG at admission; CKD = chronic kidney disease; LM = left main artery; CK-MB = creatine kinase MB isoform; CABG = coronary artery bypass grafting; ARB = angiotensin receptor blockers; OAC = oral anticoagulant. * In patients with no medical history of previous AF. ** New or presumably new BBB. *** All patients were discharged from hospital with dual antiplatelet therapy, and the mean duration of dual antiplatelet therapy was 9 ± 3 months.

**Table 2:** The incidence and the individual components of MACE in stable patients during follow-up.

	At 2 Years	At 4 Years	At 6 Years	At 8 Years
MACE, *n* (%)	36(1.3)	66(2.4)	102(3.7)	107(3.9)
Death, *n* (%)	18(0.6)	36(1.3)	52(1.9)	57(2.1)
Non-fatal recurrent infarction, *n* (%)	12(0.4)	27(1.0)	45(1.7)	45(1.6)
TVR, *n* (%)	15(0.5)	28(1.1)	45(1.7)	47(1.8)
Non-fatal stroke, *n* (%)	3(0.01)	11(0.4)	12(0.4)	14(0.5)

MACE = major adverse cardiovascular events; TVR = target vessel revascularization.

**Table 3:** Predictors for eight years MACE (Cox regression model) in stable patients.

	Univariable Analysis		Multivariable Analysis	
	HR (95%CI)	*p* Value	HR (95%CI)	*p* Value
Age > 60 years	2.10 (1.09–3.05)	0.001	2.07 (1.20–2.51)	0.010
*Age 60–69* vs. *<60 years*	*1.68 (1.03–2.64)*	*0.031*	*1.65 (1.06–2.37)*	*0.026*
*Age 70–79* vs. *<60 years*	*1.85 (1.06–3.25)*	*0.033*	*1.82 (1.05–3.20)*	*0.032*
*Age ≥80* vs. *<60 years*	*3.51 (1.28–6.99)*	*0.010*	*3.16 (1.11–9.65)*	*0.032*
EF < 50%	2.49 (1.55–3.82)	<0.001	2.47 (1.52–4.10)	<0.001
*EF < 40%* vs. *EF ≥ 50%*	*2.56 (1.54–4.90)*	*<0.001*	*2.38 (1.36–3.69)*	*0.002*
*EF 40–49%* vs. *EF ≥ 50%*	*2.42 (1.67–4.15)*	*<0.001*	*2.32 (1.31–4.13)*	*0.004*
Amiodarone (ongoing therapy)	1.72 (1.05–1.97)	0.044		
Diabetes	1.71 (1.14–2.67)	0.010	1.49 (1.09–2.31)	0.049
Diuretics (ongoing therapy)	1.69 (1.24–2.13)	0.040		
3-vessel disease	1.68 (1.12–2.52)	0.011	1.44 (1.06–2.43)	0.048
Glycemia at admission	1.02 (1.01–1.03)	0.010		
Previous stroke	1.58 (1.01–2.76)	0.044		
New-onset AF	1.50 (0.86–1.67)	0.117		
Troponin	1.01 (0.98–1.02)	0.354		

**Table 4:** Sensitivity, specificity, positive predictive value (PPV), and negative predictive value (NPV) for each predictor.

	Sensitivity	Specificity	PPV	NPV
Age > 60 years	64.8%	64.5%	14.4%	97.3%
EF < 50%	62.8%	69.5%	25.6%	98.1%
3-vessel disease	51.2%	75.5%	14.3%	96.9%
Diabetes	50.9%	82%	15.5%	97%

## Data Availability

The data presented in this study are available on request from the corresponding author due to ethical reasons.
